# Progressive contraction of the latent HIV reservoir around a core of less-differentiated CD4^+^ memory T Cells

**DOI:** 10.1038/ncomms6407

**Published:** 2014-11-10

**Authors:** S. Jaafoura, M. G. de Goër de Herve, E. A. Hernandez-Vargas, H. Hendel-Chavez, M. Abdoh, M. C. Mateo, R. Krzysiek, M. Merad, R. Seng, M. Tardieu, J. F. Delfraissy, C. Goujard, Y. Taoufik

**Affiliations:** 1 INSERM U1012, 94276 Le Kremlin-Bicêtre, France; 2 Systems Medicine of Infectious Diseases, Helmholtz Centre for Infection Research, Inhoffenstraße 7, 38124 Braunschweig, Germany; 3 INSERM U996, 92140 Clamart, France; 4 Faculté de Médecine, Université Paris-Sud, 94276 Le Kremlin-Bicêtre, France; 5 Department of Medicine, Institut Gustave Roussy, 94805 Villejuif, France; 6 INSERM U1018, 94275 Le Kremlin-Bicêtre, France; 7 Department of Internal Medicine, Hôpitaux Universitaires Paris-Sud, 94275 Le Kremlin-Bicêtre, France

## Abstract

In patients who are receiving prolonged antiretroviral treatment (ART), HIV can persist within a small pool of long-lived resting memory CD4^+^ T cells infected with integrated latent virus. This latent reservoir involves distinct memory subsets. Here we provide results that suggest a progressive reduction of the size of the blood latent reservoir around a core of less-differentiated memory subsets (central memory and stem cell-like memory (T_SCM_) CD4^+^ T cells). This process appears to be driven by the differences in initial sizes and decay rates between latently infected memory subsets. Our results also suggest an extreme stability of the T_SCM_ sub-reservoir, the size of which is directly related to cumulative plasma virus exposure before the onset of ART, stressing the importance of early initiation of effective ART. The presence of these intrinsic dynamics within the latent reservoir may have implications for the design of optimal HIV therapeutic purging strategies.

## Abstract (web summary)

HIV can persist in CD4^+^ T cells of patients receiving long-term antiretroviral therapy. Here the authors show the presence of intrinsic dynamics that progressively contract the latent HIV reservoir around a core of less-differentiated CD4 T-cell memory subsets.

## Graphical abstract

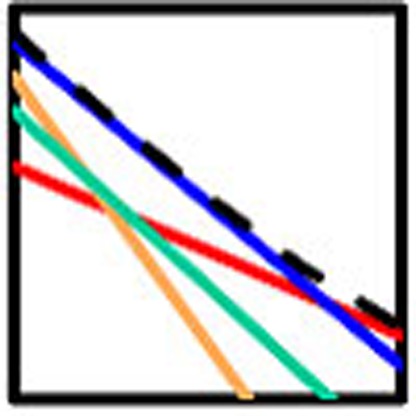

Although combined antiretroviral therapy (ART) generally suppresses HIV replication to undetectable plasma levels for prolonged periods, it fails to eradicate the virus. HIV can persist within a small pool of long-lived resting memory CD4^+^ T cells infected with integrated latent virus^1^,^2^,^3^,^4^. This latent reservoir appears to involve several memory CD4^+^ T-cell subsets at distinct differentiation stages with different phenotypic and functional properties, forming distinct ‘sub-reservoirs’^5^,^6^. Precise immunological characterization of the latent CD4^+^ T-cell reservoir, including the size of each sub-reservoir, is crucially important for the complex challenge of ‘therapeutic purging’. The relative size of each sub-reservoir may depend on its decay rate and may therefore vary according to the time on ART.

Here we show the existence of a dynamic process that progressively reduces the size of the latent reservoir around a core of less differentiated memory CD4 T-cell subsets (for example, central memory CD4^+^ T cells and the recently identified stem cell-like memory CD4^+^ T cells). Our results also stress the importance of early initiation of effective ART to limit the size of the T_SCM_ sub-reservoir, which appears directly related to cumulative plasma virus exposure.

## Results

### Study design

**Figure 1:**
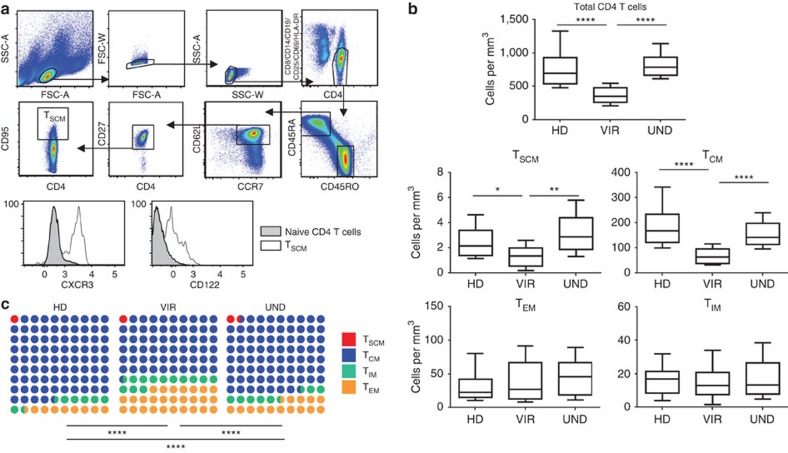
During HIV infection the relative size of the T_SCM_ subset within memory CD4 T cells remains stable. (**a**) The gating strategy used to sort the memory cell subsets: CD4-enriched PBMC were stained with a cocktail of antibodies (see Methods) and doublets were excluded on the basis of both forward scatter (FSC) and side scatter (SSC). Resting CD4^+^ T cells were gated after exclusion of CD19^+^, CD14^+^, CD8^+^, HLADR^+^, CD69^+^, CD25^+^ cells. Resting T_SCM_ were sorted on the basis of the following phenotype: CD45RA^+^ CD45RO^−^ CCR7^+^ CD62L^+^ CD27^+^ CD95^+^. CXCR3 and CD122 expression by T_SCM_ is also shown. (**b**) The absolute number of cells in each memory subset (T_SCM_, T_CM_, T_EM_ and T_IM_) was determined in HIV-infected patients with undetectable plasma viral load on ART and with CD4 cell counts above 500 per mm^3^, who were tested for integrated virus (UND, *n*=38), as well as in age- and sex-matched viremic patients (VIR, *n*=18) and HIV-seronegative healthy donors (HD, *n*=20). The Kruskal–Wallis and Dunn tests were used for statistical analysis (*P<0.05, **P<0.01, ****P<0.0001). Boxes represent the median and the 25th and 75th percentiles; whiskers represent the 10th and 90th percentiles. (**c**) Shows the percentage of each memory subset within the compartment formed by the four memory subsets analysed. The *χ*^2^ test was used to analyse differences in the distribution of the four memory subsets (****P<0.0001).

We examined the decay rates of resting memory subsets latently infected by HIV in highly selected patients with consistently undetectable plasma virus on ART. Cell sorting of CD4^+^ T-cell memory subsets requires the use of fresh peripheral blood mononuclear cells (PBMC), as cryopreservation alters the expression of markers, such as CD62L, which is required to sort the different memory subsets^7^,^8^ (see also Supplementary Fig. 1). This ruled out a retrospective study on frozen cells. Furthermore, a longitudinal prospective study can take more than a decade. We therefore chose to conduct a cross-sectional analysis on strictly selected patients from a cohort of 360 HIV-1-infected patients (see methods). The characteristics of patients who fulfilled the selection criteria and were enrolled are shown in Supplementary Table 1. The recently identified T_SCM_ subset consists of rare CD4^+^ memory T cells with stem cell-like features^9^. In response to recall antigens, T_SCM_ exhibited the strongest proliferation among the tested memory cell subsets (Supplementary Fig. 2). Figure 1a shows the gating strategy used to sort highly purified resting T_SCM_ (see also Supplementary Fig. 3 for purity). Resting central memory (T_CM_) and effector memory CD4^+^ T cells (T_EM_) were sorted on the basis of stringent criteria^9^. An additional resting CD4^+^ T-cell memory subset with an intermediate CCR7^−^ CD62L^+^ phenotype, designated T_IM_ (intermediate memory), was also sorted (see Methods). Infectious virus was recovered from the four resting memory cell subsets following *in vitro* activation (see Supplementary Fig. 4).

### Tight homeostatic regulation of T_SCM_

Cell numbers of the different memory subsets per mm^3^ of blood were similar in the patients with undetectable plasma virus and in a group of HIV-seronegative healthy donors (Fig. 1b). In contrast, in a group of patients with chronic active viral replication, the T_SCM_ and T_CM_ CD4^+^ subsets were significantly depleted (Fig. 1b). Within the pool of memory CD4^+^ T cells, the distribution of the different memory subsets was markedly different between patients with chronic active HIV replication and HIV-seronegative donors (Fig. 1c). This was mainly due to a significant increase in the proportions of T_EM_ and T_IM_, to the detriment of T_CM_ (*P*<0.01 each for the percentages of T_CM_, T_EM_ and T_IM_ within the pool of memory CD4^+^ T cells; Kruskal–Wallis and Dunn’s tests; see Supplementary Fig. 5). Possible explanations for these differences include increased differentiation of T_CM_ towards memory effector subsets, higher susceptibility of T_CM_ to infection-induced cell death and altered T_CM_ trafficking between secondary lymphoid tissues and blood. In the patients with undetectable plasma virus on ART, the percentages of T_CM_ and T_EM_ within the pool of memory CD4^+^ T cells were significantly different when compared with healthy donors (*P*<0.01; Kruskal–Wallis and Dunn’s tests), but were not different when compared with patients with active virus replication (see Supplementary Fig. 5). The percentage of T_IM_ was not different between patients on ART with undetectable virus and both healthy donors and viremic patients. As shown in Fig. 1c, the distribution of the different subsets within memory CD4^+^ T cells differed between the patients with undetectable plasma virus on ART and both the patients with active virus replication and the healthy donors. This suggested that recovery of the CD4^+^ T-cell memory pool during prolonged effective ART was incomplete. Interestingly, there was no significant difference in the percentage of T_SCM_ within memory CD4^+^ T cells between the patients on prolonged effective ART, the patients with active virus replication and the healthy donors (see Supplementary Fig. 5). This suggests tight homeostatic regulation maintaining the equilibrium between T_SCM_ and the rest of the memory CD4^+^ T cells despite active virus replication. In each group of patients and healthy donors analysed, there was no significant relation between the age and the absolute numbers of T_SCM_ per mm^3^ of blood (*P*=0.12, 0.53 and 0.69, for patients with chronic active virus replication, patients on prolonged effective ART and healthy donors, respectively, Spearman Rank Test) or the percentage of T_SCM_ within memory CD4^+^ T cell (*P*=0.83, 0.11 and 0.11, respectively, Spearman Rank Test).

### A highly stable Core of latently infected T_SCM_ and T_CM_

**Figure 2:**
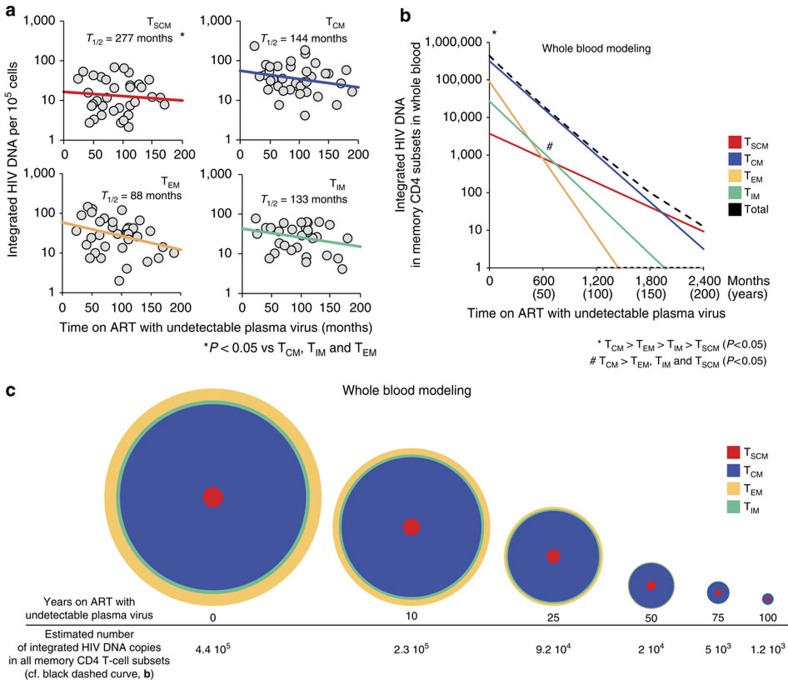
Slow reduction of the latent HIV reservoir around a core formed mainly by T_SCM_ and T_CM_. (**a**) Integrated HIV DNA was quantified in T_SCM_, T_CM_, T_EM_ and T_IM_ isolated from 38 strictly selected patients in whom viral load was undetectable for 24–189 months on ART. Recovery of T_EM_ and T_SCM_ was suboptimal in three patients. T_IM_ could not be isolated from nine patients. The results are expressed as the integrated HIV DNA copy number per 10^5^ cells. Taking inter-patient variability into account, the data shown in **a** were analysed with the Monte Carlo algorithm with 10^6^ computer simulations with random samplings for each latently infected memory subset (see Methods). For each simulation, the number of integrated HIV DNA copies per 10^5^ cells at the time of undetectable plasma virus following ART initiation (*y*_*0*_) and the slope of decay (*λ*) were fitted. The distributions of *y*_*0*_ and *λ* obtained from the 10^6^ simulations are shown in Supplementary Fig. 8. The mean values of *y*_*0*_ and *λ* were used to plot the decay curves shown in **a**. The mean half-life of decay (−ln2/*λ*) is indicated for each latently infected memory subset. Statistical significance is indicated as * (for statistical analysis, see the Monte Carlo section of Methods). (**b**) Extends the data shown in **a** to the entire blood compartment. To analyse the decay in absolute numbers of latently infected memory subsets in the whole blood compartment, we used the 10^6^ simulation values of *y*_0_ and slopes of decay obtained for each memory subset following Monte Carlo analysis. The predictions shown in **b** correspond to the average values of *y*_*0*_ and slopes. For the other parameters required to calculate the absolute numbers of latently infected cells (body weight, CD4 T-cell count and the percentages of the memory subsets among total CD4 T cells), we used the median values from our cohort of 38 patients. Total blood volume was estimated to represent 7% of total body weight^29^. Note that the predictions in **b** remained consistent when random sampling (Monte Carlo approach) was used for body weight, the CD4^+^ T-cell count and the percentages of memory subsets among total CD4^+^ T cells, based on the median values and variance for the patients analysed. We considered 50 time points from 0 to 200 years (a total of 50 × 10^6^ values for each memory subset). Comparison between the memory subsets was performed as in **a**. (See in Methods the Monte Carlo section). Statistical significance is indicated *, ^#^. (**c**) The immunological dynamics of the CD4^+^ T-cell latent reservoir, corresponding to changes, at chosen time-points, in the relative size of each memory subset within the compartment formed by the four latently infected memory subsets in the entire blood compartment (see above). The surface areas are proportional to the numbers of integrated HIV DNA copies in each memory CD4 subset in the entire blood volume (predictions shown in **b**).

Integrated HIV DNA was quantified in sorted CD4 memory subsets with a well-established Alu-gag PCR method^10^,^11^ (see Methods and Supplementary Fig. 6). Sorted naive CD4^+^ T cells (CD45RA^+^ CD45RO^−^ CCR7^+^ CD62L^+^ CD27^+^ CD95^−^) were also tested, but in our series integrated HIV DNA was detected in truly naive cells only in 18.4% of patients (see Supplementary Fig. 7). The results obtained for memory subsets are shown in Fig. 2a. The best linear regression of our data suggested that the T_SCM_ sub-reservoir had the longest half-life among the latently infected memory subsets analysed (not shown). However, to take possible inter-patient variability into account and to validate this cross-sectional analysis, the results were analysed with the Monte Carlo (MC) algorithm (see Methods). Briefly, we performed 10^6^ computer simulations with random samplings for each latently infected memory subset, based on the mean values and variance of our experimental data. For each simulation, we fitted the number of integrated HIV DNA copies per 10^5^ cells at the time of undetectable plasma virus following ART initiation (*y*_*0*_) and the slope of decay (*λ*). The mean values of *y*_*0*_ and *λ* obtained from the 10^6^ simulations were used to plot the decay curves shown in Fig. 2a. The distribution of *y*_0_ and *λ* for each latently infected memory subset is indicated in Supplementary Fig. 8. The mean value of *λ* was used to estimate the half-life (−ln2/*λ*) of each latent sub-reservoir. The mean *y*_*0*_, *λ* and half-life values were as follows: 16.54 integrated copies per 10^5^ cells, −0.0025 month^−1^ and 277 months for T_SCM_; 55.54 integrated copies per 10^5^ cells, −0.0048 month^−1^ and 144 months for T_CM_; 42.92 integrated copies per 10^5^ cells, −0.0052 month^−1^ and 133 months for T_IM_ and 59.88 integrated copies per 10^5^ cells, −0.0079 month^−1^ and 88 months for T_EM_. Analysis of the MC results (two-way analysis of variance and *t*-test (significance at *P*<0.05), with a Bonferroni post-test) confirmed that the T_SCM_ latent reservoir had a slower decay rate than the other studied sub-reservoirs (*P*<0.05), whereas no significant difference was found between the decay rates of T_CM_, T_IM_ and T_EM_ (see also Supplementary Fig. 8a). The same analysis also showed that T_SCM_ had a lower number of integrated copies than the other sub-reservoirs at the time of undetectable plasma virus following ART initiation (*y*_*0*_; *P*<0.05), whereas no significant difference was found between T_CM_, T_IM_ and T_EM_ (see also Supplementary Fig. 8b).

We then predicted the individual decay of each latently infected memory subset within the entire blood volume (Fig. 2b), as well as long-term changes in the relative proportions of the different memory subsets forming the latent CD4^+^ T-cell reservoir (Fig. 2c). For this purpose, we took into account the *y*_0_ and decay rate values of each sub-reservoir obtained by MC analysis, together with the absolute number of cells composing each CD4^+^ memory subset in the entire blood volume (see Methods and legend to Fig. 2b,c). We found the following hierarchy in the total sizes of the different memory sub-reservoirs in the entire blood volume at the time of undetectable plasma virus following ART initiation: T_CM_>T_EM_>T_IM_>T_SCM_ (*P*>0.05; Fig. 2b). The relative size of the T_SCM_ sub-reservoir within the latent memory reservoir then increased over time, owing to the longer half-life of these cells (Fig. 2b,c). The data predicted that the size difference between T_SCM_, T_EM_ and T_IM_ sub-reservoirs would become non-significant after 50 years on effective ART, whereas the T_CM_ sub-reservoir would remain larger than the other three sub-reservoirs (Fig. 2b). The decay of the whole-blood latent CD4^+^ T-cell memory reservoir appeared to be driven mainly by the decay rate of the T_CM_ sub-reservoir (Fig. 2b). The results also imply that it would take more than 100 years for T_EM_, the most labile latent sub-reservoir, to be eradicated by ART, without additional therapeutic approach (Fig. 2b). Altogether, T_SCM_ and T_CM_ appear to form the most stable part of the latent CD4^+^ T-cell memory reservoir, mainly owing to the extremely long half-life of T_SCM_ and to the larger size of the T_CM_ reservoir at the initiation of effective ART (Fig. 2b,c).

### T_SCM_ reservoir size is related to cumulative HIV exposure

**Figure 3:**
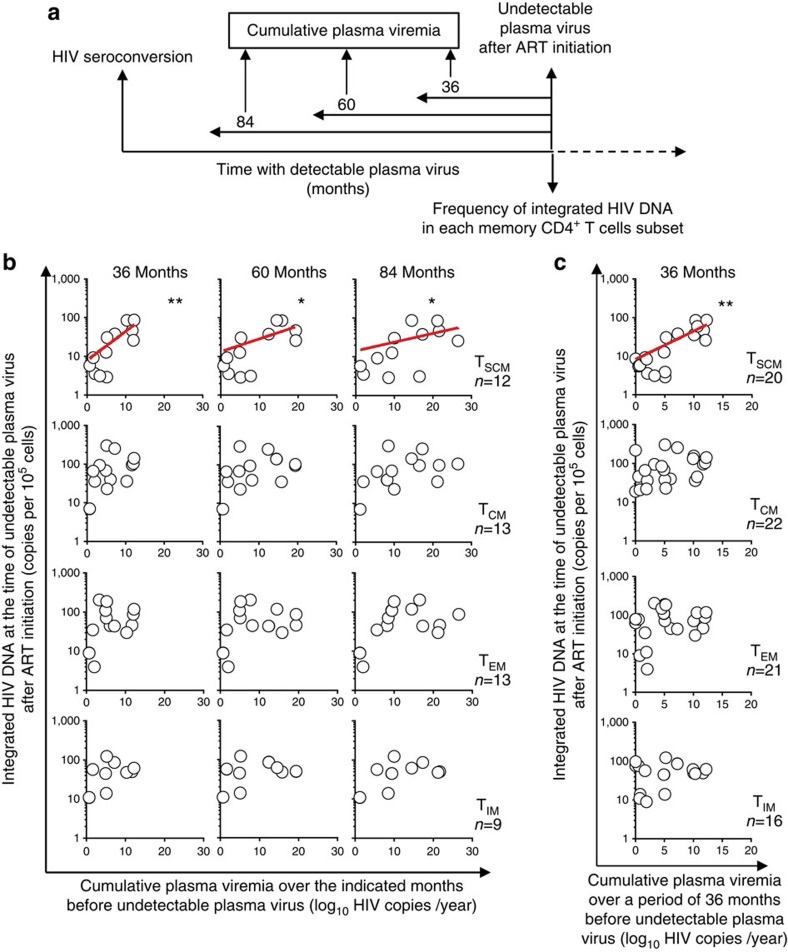
The frequency of latent infection of T_SCM_ is related to cumulative plasma virus exposure. (**a**,**b**) Cumulative viremia, expressed as log_10_ virus copy-years during periods of 36, 60 and 84 months before plasma virus became undetectable, was determined in patients (*n*=9–13) for whom virological follow-up was available for a period of 84 months. Associations were sought between cumulative viremia and the frequency of latent infection of each memory subset at the time when plasma virus became undetectable after ART initiation. For each patient, the frequency of latent infection of each memory subset at the time when plasma virus became undetectable after ART initiation was calculated from the slopes of the decay curves shown in Fig. 2a. The number of patients for whom virological follow-up was available over a period longer than 84 months before undetectable plasma virus, was insufficient for statistical analysis. In **c**, the same analysis was performed for all patients (*n*=16–22) for whom virological follow-up was available over a period of 36 months before plasma virus became undetectable. The Spearman rank test was used for statistical analysis (*P<0.05, **P<0.01).

We then examined the influence of several parameters on the frequency of integrated HIV in each memory CD4^+^ T-cell subset at the time when plasma virus became undetectable (see legend of Fig. 3), including age and sex, the CD4^+^ T-cell nadir, the duration of detectable HIV viremia and cumulative HIV plasma viremia. Cumulative plasma viremia, expressed in log_10_ virus copy-years, is a measure of cumulative plasma viral load exposure during a given time period^12^. As shown in Fig. 3a–c, the frequency of integrated HIV DNA in T_SCM_ correlated positively with cumulative HIV plasma viremia, over a period up to 7 years before plasma virus becoming undetectable, whereas no such relation was found for the other memory subsets (Fig. 3b,c). None of the other parameters significantly influenced the size of the latently infected memory subsets (*P* values ranged to from 0.18–0.9, Spearman rank test). Together, the results suggested that the size of the T_SCM_ sub-reservoir is more directly influenced by the level and duration of plasma virus exposure than the other sub-reservoirs.

## Discussion

Together, these results reveal the existence of an intrinsic dynamic process within the latent reservoir that contracts around a core of less-differentiated memory subsets (T_CM_ and T_SCM_). This slow process is driven by differences in initial sizes and decay rates of the latently infected memory subsets. The half-life of each latently infected subset may be related to the intrinsic capacity of its component cells to survive and to self-renew. Several lines of evidence suggest that homeostatic proliferation, including in response to IL-7, may not necessarily lead to efficient reactivation of latent HIV and subsequent cell destruction, thus allowing self-renewing cells and their progeny to persist in the latent CD4^+^ T-cell reservoir^5^,^13^,^14^. Long-term survival and self-renewal are key properties of T_SCM_ and, to a lesser extent, of T_CM_^9^. These features may explain the greater stability of the T_SCM_ reservoir as compared with the more highly differentiated CD4^+^ T-cell memory subsets. However, the half-lives of latently infected memory subsets may also be influenced by cell transfer from other compartments. For example, in response to homeostatic signals, T_SCM_ can generate more highly differentiated cells such as T_CM_ and T_EM_ while maintaining their own pool^9^,^15^, thereby fueling downstream compartments.

A recent work suggested that T_SCM_ harbour the highest level of HIV DNA among memory T-cell subsets regardless of the time on ART, with a considerable inter-individual variations^16^. Our results do not support this finding. We found that the T_SCM_ sub-reservoir has great stability but at the time when plasma virus became undetectable on ART, it harbours the lowest level of stable infection among latently infected memory subsets. Noteworthy, the authors used a PCR procedure that quantifies total HIV DNA including integrated and unintegrated labile virus. The latter may reflect residual virus expression^17^,^18^ that may increase inter-individual variability of total HIV DNA.

**Figure 4:**
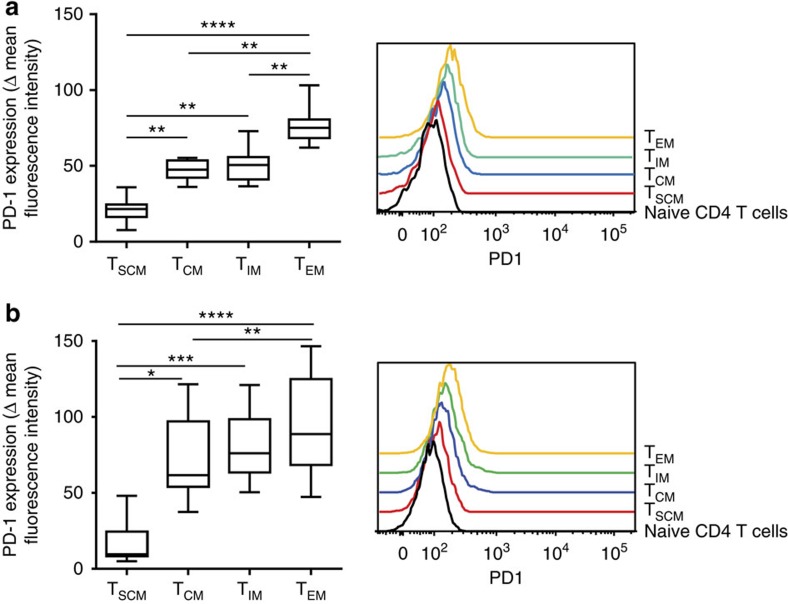
PD-1 is less strongly expressed on T_SCM_. Surface PD-1 expression on blood memory CD4^+^ T-cell subsets was analysed in 15 patients with CD4+ T-cell counts >500 per mm^3^ and undetectable plasma virus for at least 24 months on ART (**a**) and in 15 viremic patients (median CD4 T-cell count=477 per mm^3^, median viral load=3.3 log10, **b**). Data are expressed as the difference in PD-1 mean fluorescence intensity (ΔMFI) relative to naive CD4+ T cells. Boxes represent the median and the 25th and 75th percentiles; whiskers represent the 10th and 90th percentiles. Representative PD-1 staining is shown. The data were analysed with the Friedman test for paired data and with Dunn’s multiple comparison test (*P<0.05, **P<0.01, ***P<0.001, ****P<0.0001).

Why does cumulative plasma virus exposure influence the formation of the latent T_SCM_ sub-reservoir more strongly than the formation of other memory sub-reservoirs? One possibility is the existence of additional factors that influence the formation of latently infected CD4^+^ T cells in addition to viral exposure and that are more active in T_EM_, T_IM_ and T_CM_. These might include immunological factors that negatively regulate cell activation, such as cell-surface inhibitory receptors. The level of PD-1 expression has been shown to influence the level of latent infection in CD4 T cells in patients on ART^5^. As shown in Fig. 4a,b, a lower PD-1 expression was found on T_SCM_ as compared with other CD4 memory subsets in viremic patients and patients on ART with undetectable plasma virus. This was consistent with previous reports suggesting that in CD4 and CD8 T cells the expression of the inhibitory receptor PD-1 could be related to the stage of differentiation^5^,^19^,^20^. PD-1 could represent a potential target to facilitate HIV eradication^5^,^21^. However, the low PD-1 expression on T_SCM_ argues against an effectiveness of PD-1 targeting therapies in reducing the size of the T_SCM_ sub-reservoir. Alternatively, the presence within a memory subset of latently infected cells generated by indirect infection, for example, through cell transfer from the T_SCM_ pool towards more highly differentiated memory subsets, might also alter the direct relationship between the size of the sub-reservoir and cumulative viral exposure.

Our results show that it is unrealistic to expect ART alone to purge the latent HIV reservoir. PCR procedures for detecting integrated HIV may overestimate the size of the latent CD4^+^ T-cell reservoir. Indeed, a significant proportion of integrated viruses is assumed to be defective because it cannot be induced by *in vitro* activation. Importantly, the stimuli used *in vitro* to activate cells and to recover latent virus in outgrowth assays may not fully reproduce the complex activation conditions existing *in vivo*, particularly given the marked immunological heterogeneity of the latent reservoir cells. Indeed, this reservoir comprises different CD4^+^ T-cell subsets with distinct activation requirements (for instance, see Fig. 4a,b, showing variable expression of the inhibitory receptor PD-1 by different cell subsets). This implies that some latent viruses capable of generating infective virions *in vivo* may not be recoverable *in vitro*. Also, a recent elegant study showed that the latent reservoir contains proviruses with no deletions or inactivating mutations that are not induced after *in vitro* activation, but might possibly be recoverable *in vivo*^22^. The authors of this latter study forwarded a model of stochastic induction of intact proviruses following cell activation^22^. Clearly, although PCR procedures for detecting integrated HIV may overestimate the size of the latent reservoir, viral outgrowth assays can underestimate it^22^. In practice, this means that a patient cannot be said to be cured of HIV infection unless integrated virus is undetectable by PCR.

The part of the latent CD4 T-cell reservoir formed by T_CM_ and T_SCM_ should be a priority target for therapeutic strategies addressing the latent reservoir. Therapeutic approaches targeting key memory and effector differentiation pathways could also help to drive latently infected memory cells, beyond the effector memory state, towards short-lived terminally differentiated effector states, thereby accelerating the decay of the latent reservoir. Our results also stress the importance of early initiation of effective ART to limit the size of the T_SCM_ reservoir, which appears directly related to cumulative plasma virus exposure.

## Methods

### Patients

Patients were selected from a cohort of 360 HIV-1-infected patients in whom plasma viral load was determined every 3 months. To be eligible for the study, patients had to have undetectable plasma virus on combined antiretroviral therapy (ART) for at least 24 months. Detection of a single viral blip was a formal exclusion criterion. Patients also had to have a CD4^+^ T-cell count higher than 500 per mm^3^ of blood, indicating effective immune recovery on ART, at the time when their cell subsets were tested for integrated HIV or for viral production. Forty-five eligible patients (plasma virus load undetectable (<20 copies per ml) for 24–189 months) agreed to participate in this cross-sectional study. Integrated HIV DNA was quantified in 38 patients. Characteristics of the patients are indicated in Supplementary Table 1. Cells from seven patients with undetectable plasma virus for 34–148 months were used for inducing virus production *in vitro*. Written informed consent was obtained from each patient and the study was approved by the local ethics committee (Comité pour la Protection des Personnes Ile-de-France VII, (CPP IDF VII) Bicêtre, France). Cumulative HIV plasma viremia up to the time when plasma virus became undetectable (from 12–84 months before than plasma virus became undetectable) was determined as previously described^13^ in patients for whom plasma viral load data were available.

### Flow cytometry

A blood sample of 200 μl was used for CD4^+^ memory T-cell phenotyping with the following antibodies: anti-CD8-FITC (1/10, clone RPA-T8), anti-PD1-FITC (1/5, clone MIH4), anti-CD122-PE (1/10, clone Mik-B3), anti-CD62L-V450 (1/10, clone DREG-56), anti-CD4-V500 (1/20, clone RPA-T4), anti-CD95-APC (1/10, clone DX2), anti-CD45RA-PE-Cy7 (1/20, clone HI100), anti-CD45RO-PerCPCy5.5 (1/10, clone UCHL1), anti-CCR7-PE-CF594 (1/10, clone 150503), anti-CXCR3-Alexa 700 (1/10, clone 1C6/CXCR3), anti-CD27-APC-H7 (1/10, clone M-T271) (all from BD Biosciences), and anti-CD3-eFluor 650NC (1/10, clone OKT3, eBioscience). After staining, the blood sample was fixed (fix/lyse solution, BD Biosciences) and cells were acquired on a BD LSR Fortessa cytometer (BD Biosciences). Data were analysed with Flow Jo software.

### Cell sorting

CD4^+^ T cells were enriched from PBMC by negative selection with magnetic beads (CD4 BD Imag, BD Biosciences). The cells were then stained with a cocktail of the following antibodies: anti-HLA-DR-FITC (1/15, clone G46-6), anti-CD8 FITC (1/15, clone RPA-T8), anti-CD19-FITC (1/15, clone HIB19), anti-CD14 FITC (1/15, clone M5E2), anti-CD25-FITC (1/6, clone M-A251), anti-CD69-FITC (1/6, clone FN50), anti-CD3-Alexa 700 (1/15, clone OKT3), anti-CD122-PE (1/12, clone Mik-B3), anti-CD62L-V450 (1/15, clone DREG-56), anti-CD4-V500 (1/30, clone RPA-T4), anti-CD95-APC (1/15, clone DX2), anti-CD45RA-PE-Cy7 (1/30, clone HI100), anti-CD45RO-PerCP-Cy5.5 (1/15, clone UCHL1), anti-CCR7-PE-CF594 (1/15, clone 150503) and anti-CD27-APC-H7 (1/30, clone M-T271) (all from BD Biosciences), then sorted with a BD FacS ARIA III cytometer (BD Biosciences). Non-CD4^+^ T cells and possibly activated CD4^+^ T cells were gated out by fluorescein isothiocyanate staining. After gating on the live-cell gate and doublet exclusion, highly purified memory CD4^+^ T-cell subsets were sorted on the basis of the following phenotypes: (i) stem cell memory CD4^+^ T cells (T_SCM_): CD3^+^ CD4^+^ CD45RA^+^ CD45RO^−^ CCR7^+^ CD62L^+^ CD27^+^ CD95^+9^; (ii) central memory CD4^+^ T cells (T_CM_): CD3^+^ CD4^+^ CD45RA^−^ CD45RO^+^ CCR7^+^ CD62L^+^ and (iii) effector memory CD4 T cells (T_EM_): CD3^+^ CD4^+^ CD45RA^−^ CD45RO^+^ CCR7^−^ CD62L^−^. An additional subset with an intermediate phenotype (T_IM_) was also sorted: CD3^+^ CD4^+^ CD45RA^−^ CD45RO^+^ CCR7^−^ CD62L^+^.

### Latent HIV quantification

Integrated HIV DNA was quantified by two-step ALU-gag PCR as previously described^10^,^11^ (see also Supplementary Fig. 6). Serial dilutions of the 8E5 cell line (American Type Culture Collection) in PBMC from HIV-seronegative donors were used to generate a standard curve with which to quantify integrated HIV DNA (Supplementary Fig. 6a). The 8E5 cell line harbours a single integrated HIV copy per genome. The standard curve spanned 2,000 to 1 HIV copies per PCR. The PCR method was able to detect one integrated HIV DNA copy (one 8E5 cell) among 100,000 cellular genomes (Supplementary Fig. 6a). The same numbers of resting T_SCM,_ T_CM_, T_EM_ and T_IM_, corresponding to the lowest cell number recovered among the four cell subsets after sorting, were pelleted for DNA extraction. In all patients, glyceraldehyde 3-phosphate dehydrogenase quantitative PCR performed on 10% of the extracted DNA showed that similar amounts of DNA were effectively recovered from the cell subsets. Duplicate cell aliquots were used whenever possible. The product of the first Alu-gag PCR was split into five 10-μl aliquots, and each aliquot was submitted to nested PCR^10^,^11^. We used the mean value obtained for the five aliquots. When duplicate cellular aliquots were available, we used the mean of the ten values. Additional information about Latent HIV quantification is provided in Supplementary Methods.

### Statistical analysis

Statistical analysis was performed using Prism software (GraphPad). Differences between groups were analysed using the Kruskal–Wallis and Dunn’s tests for unpaired continuous variables, and the Friedman and Dunn tests for paired continuous variables. The *χ*^2^ test was used for categorical variables. The Spearman rank test was used to examine associations between the size of the latently infected memory subsets at the time when plasma virus became undetectable and parameters including age and sex, the CD4^+^ T-cell nadir, the duration of HIV viremia and cumulative HIV viremia before plasma virus becoming undetectable.

### Estimation of decay rates of CD4^+^ T-cell sub-reservoirs

Bootstrapping is a statistic method for assigning measures of accuracy to estimates^23^,^24^. A standard choice for approximating the distribution is to use the empirical distribution of the observed data. This can be implemented by constructing a number of resamples of the data set, each of which is obtained by random sampling with replacement from the original data set.

In this paper, for each CD4^+^ T-cell memory reservoir, we consider the following model:


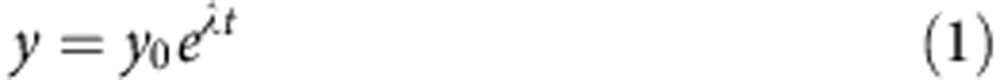


where *y*_*0*_ is the integrated HIV DNA copy number at the time of undetectable plasma virus following ART initation, *λ* is the slope of decay for each latent sub-reservoir and *t* is the duration of plasma virus undetectability. The half-life is computed with the term −ln2/*λ*. Note that we considered nonlinear mathematical models, however we could not find any statistically significant improvement.

Therefore, given a vector observation (measurement) *y*_*i*_ at time *t*_*i*_, the parameter set *φ*=[λ,*y*_0_] is computed for each CD4^+^ T-cell memory sub-reservoir (inverse problem^23^).

The parameter fitting is performed by minimizing the root mean square difference on a *log* scale between the model predictive output (*x*_*i*_) and the experimental measurement (*y*_*i*_), as follows:


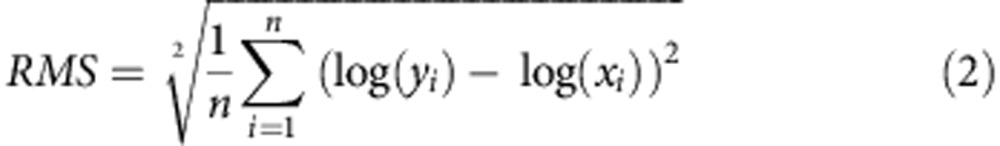


where *n* is the total number of measurements. The minimization of root mean square is performed using the differential evolution algorithm^25^. Several optimization solvers were tested, including both deterministic (fmincon Matlab routine, Threshold Acceptance algorithm and Pattern Search algorithm) and stochastic methods (Genetic algorithm and Annealing algorithm). We found that the differential evolution global optimization algorithm was robust to initial guesses of parameters and converged faster with more certainty than the other methods.

As inter-patient variability is non-negligible in this work, we considered the bootstrap method to analyse mathematical models in stochastic environments. Bootstrap is useful when there is no form to estimate the distribution of the statistic of interest^24^. One way of performing case resampling is to use the MC algorithm. This methodology has been successfully used to address different biological problems^24^,^26^.

### MC Analysis

The MC algorithm^24^,^27^ is a large dimensional-space method that uses repeated random samplings to obtain numerical results in a stochastic environment when experiments cannot be repeated several times and when variability is present.

To settle down the simulations, we resample the data with replacements. The size of the resample must be equal to the size of the original set. To this end, based on the variance of our raw data for each time point in Fig. 2a, we generated a vector with random samplings using a geometric distribution (results were consistent using normal or exponential distribution). Using equation (1) we again refitted the parameters (*λ*, *y*_0_), and repeated this process 10^6^ times for each CD4^+^ T-cell subset. This large number of repetitions provides a more precise estimate of the parameter distribution. Histograms showing the distribution of *y*_*0*_ and *λ* obtained by bootstrapping can be found in Supplementary Fig. 8. The 95% confidence interval of parameter estimates is computed using the outcome of the bootstrap method^28^. For each constant parameter, we select the 2.5 and 97.5% quantiles of the 10^6^ estimates to form the 95% confidence interval. The results are presented in Supplementary Fig. 8.

Analyses of the MC outcome were implemented with two-way analysis of variance and a *t*-test. Significance was assumed at *P*<0.05, and the data were further analysed with a Bonferroni post-test.

## Author contributions

S.J. designed and performed experiments and analysed data. M.G.deG.deH. designed and performed experiments, analysed data, performed statistical analysis and wrote the paper. E.A.H.-V. analysed data, performed mathematical modelling and statistical analysis and wrote the paper. M.A. performed experiments and analysed data. M.C.M. performed experiments and analysed data. R.K. analysed data. M.M. selected patients and analysed data. R.S. performed statistical analysis. M.T. designed experiments and analysed data. J.F.D. designed experiments and analysed data. C.G. selected patients, designed experiments and analysed results. Y.T. designed experiments, analysed data, wrote the paper and supervised the project.

## Additional information

**How to cite this article**: Jaafoura, S. *et al.* Progressive contraction of the latent HIV reservoir around a core of less-differentiated CD4^+^ memory T-Cells. *Nat. Commun.* 5:5407 doi: 10.1038/ncomms6407 (2014).

## Supplementary Material

Supplementary Information Supplementary Figures 1-8, Supplementary Table 1 and Supplementary Methods.
