# Supplementary material for: Progressive contraction of the latent HIV reservoir around a core of less-differentiated CD4+ memory T Cells
Source: Nat Commun. 2014 Nov 10;5:5407. doi: 10.1038/ncomms6407 (PMC4241984; doi:10.1038/ncomms6407)
Supplement: Supplementary Information — Supplementary Figures 1-8, Supplementary Table 1 and Supplementary Methods. [file ncomms6407-s1.pdf]

Supplementary Figure 1

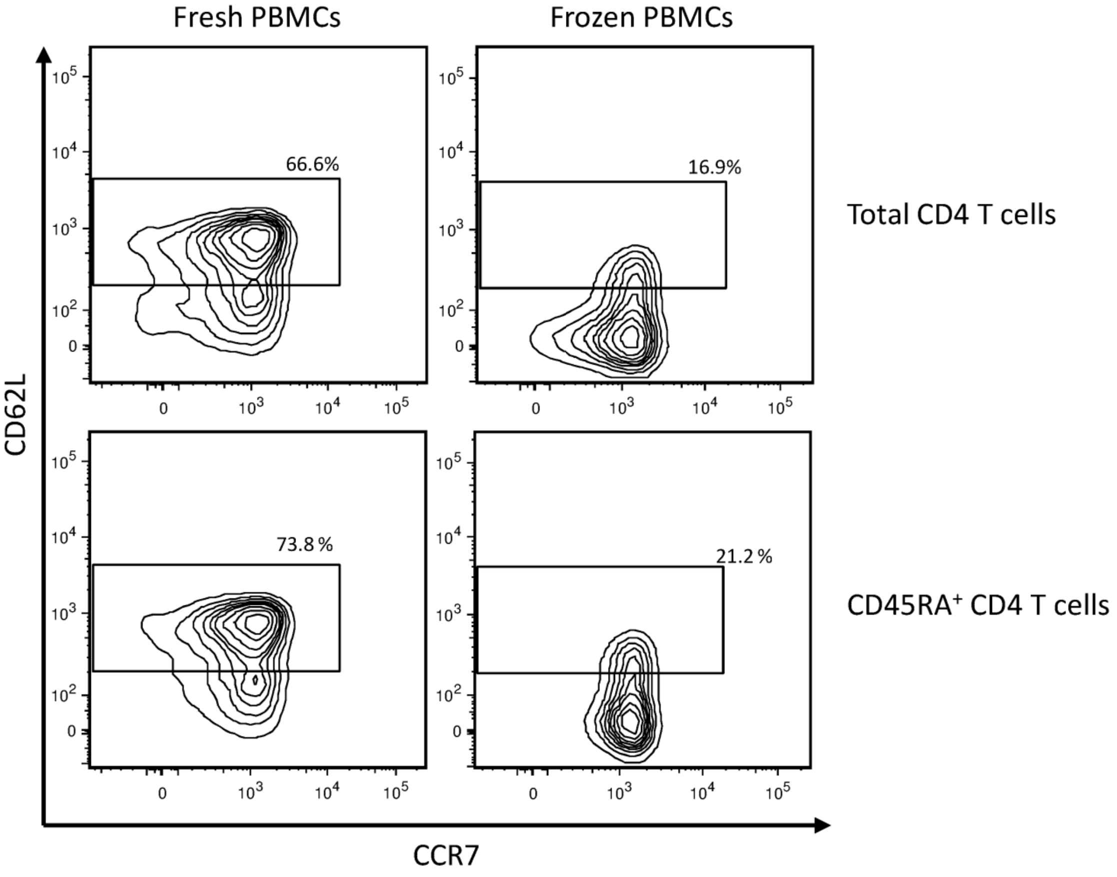

Supplementary Figure 1: Cryopreservation alters CD62L expression by CD4 T cells. Freshly isolated (left) or cryopreserved PBMCs (right) were stained with the mix of antibodies described in the cell sorting section. CCR7 and CD62L staining in CD4 T cells (top) and CD4+ CD45RA+ T cells are shown (bottom).

Supplementary Figure 2

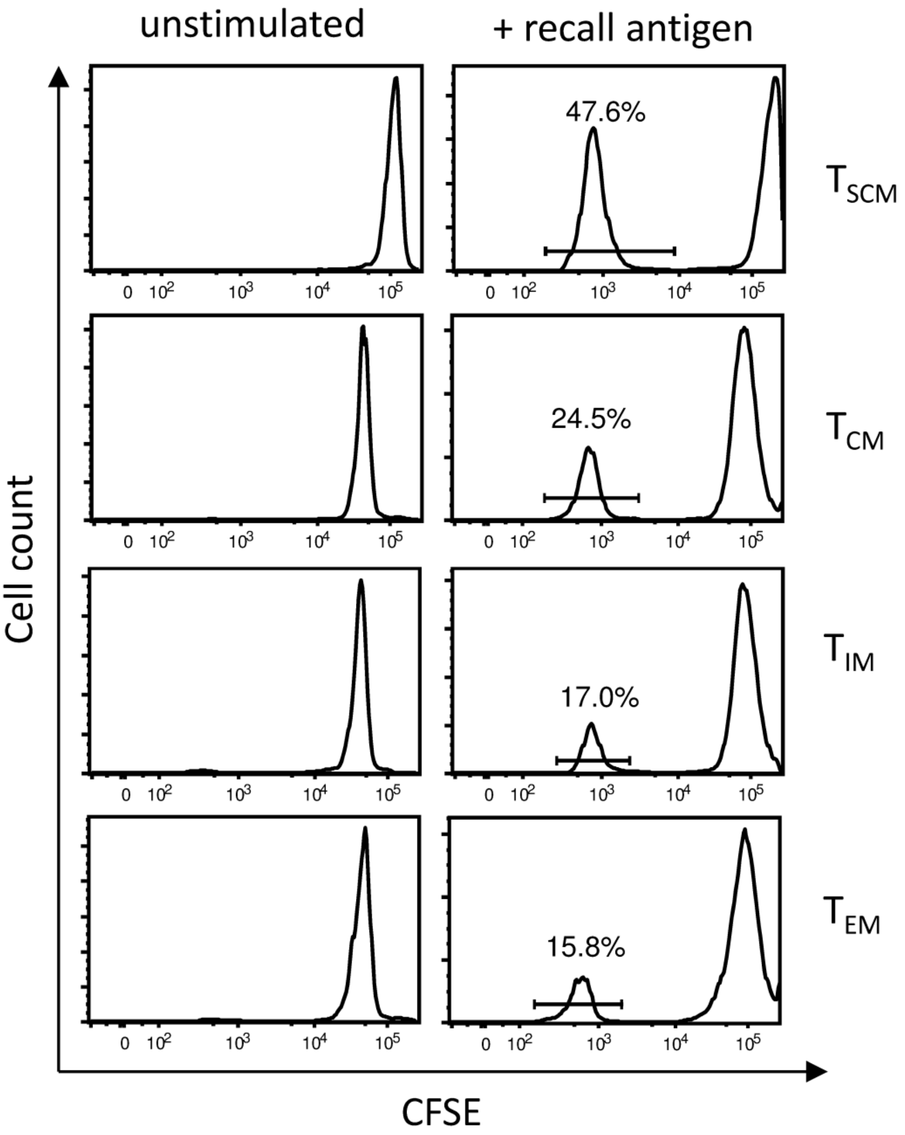

Supplementary Figure 2: Proliferation of CD4+ T cell memory subsets to recall antigens. Sorted T_{SCM}, T_{CM}, T_{EM} and T_{IM} were stained with CFSE and cultured with a mix of recall antigens (PPD, TT, VZV, HSV, CMV) in the presence of autologous CD8-depleted PBMC for 5 days (see supplementary methods). CFSE dilution was analyzed by flow cytometry in each CD4+ memory T cell subset.

Supplementary Figure 3

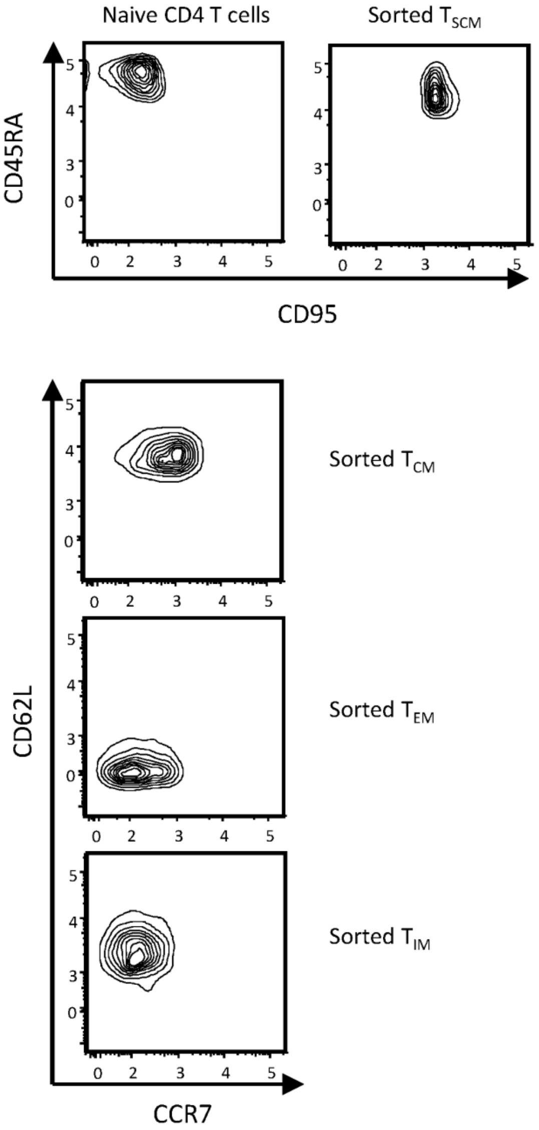

Supplementary Figure 3: Purity of sorted memory CD4+ T cell subsets. Sorted memory CD4+ T cell subsets were analyzed for purity by flow cytometry. CD95 staining is shown for naive and T_{SCM} subsets. CCR7 and CD62L staining is shown for T_{CM}, T_{EM} and T_{IM}. Purity was higher than 99.9% for each sorted CD4+ memory T cell subset.

Supplementary Figure 4

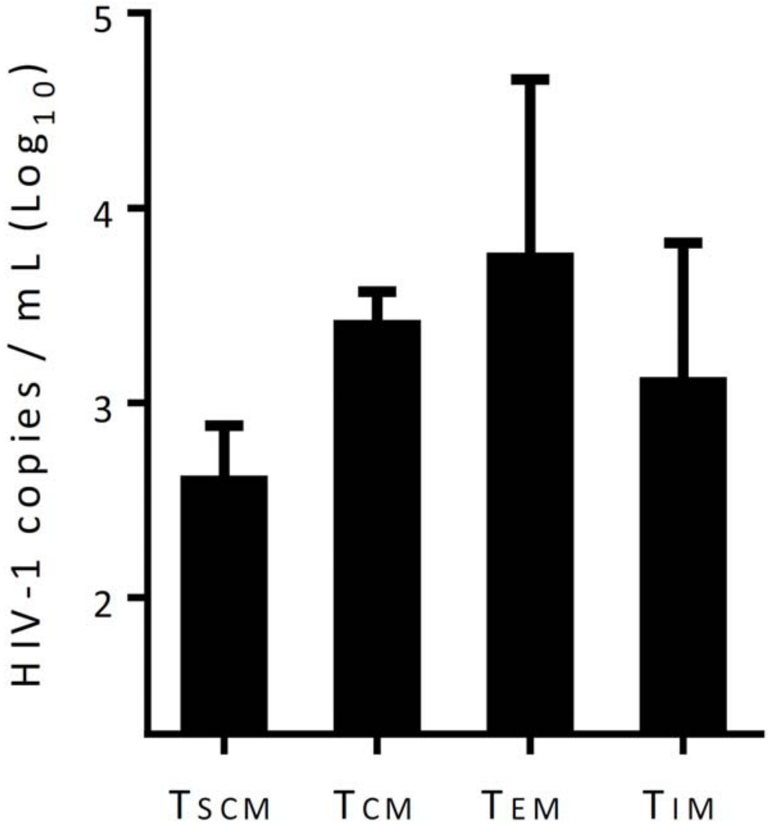

Supplementary Figure 4: Virus production by memory CD4+ T cell subsets. Sorted resting memory CD4+ T cell subsets were activated as indicated in supplementary methods. HIV RNA was quantified in the supernatants by quantitative RT-PCR on day 21. The values are the mean ± SEM of values obtained in 7 patients.

Supplementary Figure 5

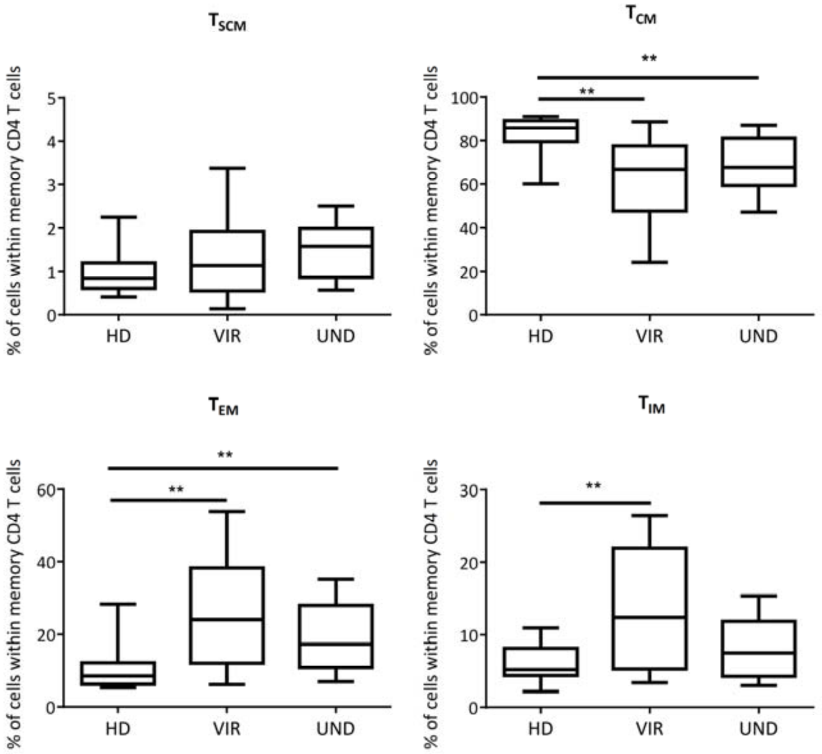

Supplementary Figure 5: During HIV infection the proportion of T_{SCM} within the pool of memory CD4 T cells remains stable. The percentage of cells of each memory subset (T_{SCM}, T_{CM}, T_{EM} and T_{IM}) within the pool of memory CD4 T cells was determined in HIV-infected patients with undetectable plasma viral load on ART and with CD4 cell counts above 500/µL, who were tested for integrated virus (UND, n=38), as well as in age- and sex-matched viremic patients (VIR, n=18) and HIV-seronegative healthy donors (HD, n=20). The Kruskal-Wallis and Dunn tests were used for statistical analysis (** $p < 0.01$). Boxes represent the median and the 25th and 75th percentiles; whiskers represent the 10th and 90th percentiles.

Supplementary Figure 6

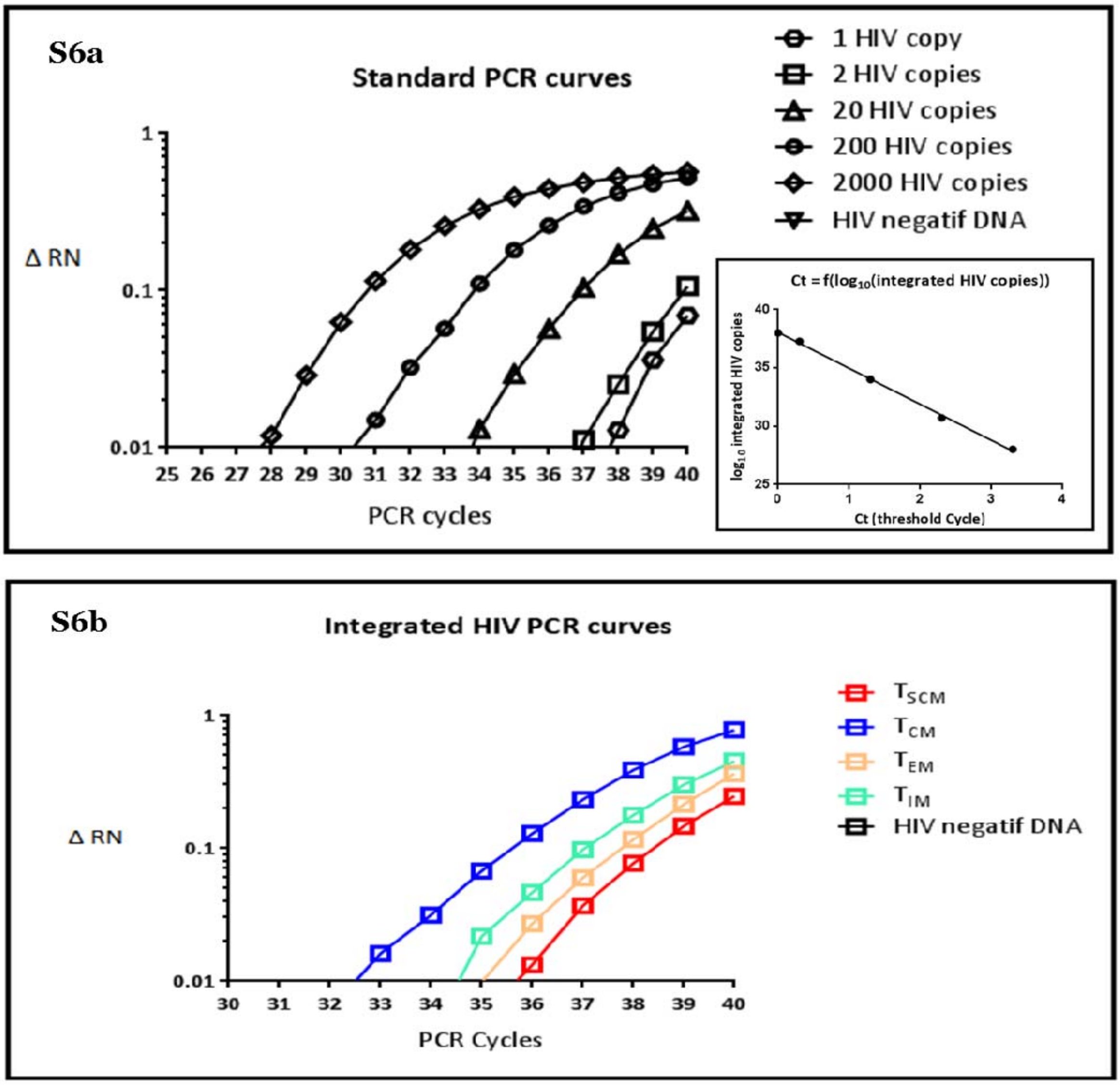

Supplementary Figure 6: Quantification of integrated HIV DNA. **S6a** shows the PCR curves [Fluorescence signal (expressed as log10 ΔRn) vs. PCR cycles] obtained after Alu-gag PCR amplification [10,11] of serial dilutions of the 8E5 cell line (ATCC) in PBMC from HIV-seronegative donors (from 2000 to 1 integrated copies per PCR). ΔRn (delta normalized reporter) = Rn of standards or samples minus Rn of 0PCR (PCR mix without DNA template). **S6b** shows ALU-gag PCR amplification of DNA from sorted memory CD4 T

cell subsets from a patient on prolonged HAART. In **S6a** and **S6b**, ∆Rn of HIV negative DNA controls were below 0.01 at the different PCR cycles. The standard curve [Ct (threshold cycle) vs. $\log_{10}$ integrated HIV copies] generated from the standard PCR curves and used to quantify the number of integrated HIV DNA in memory T cells is also shown in **S6a**.

Supplementary Figure 7

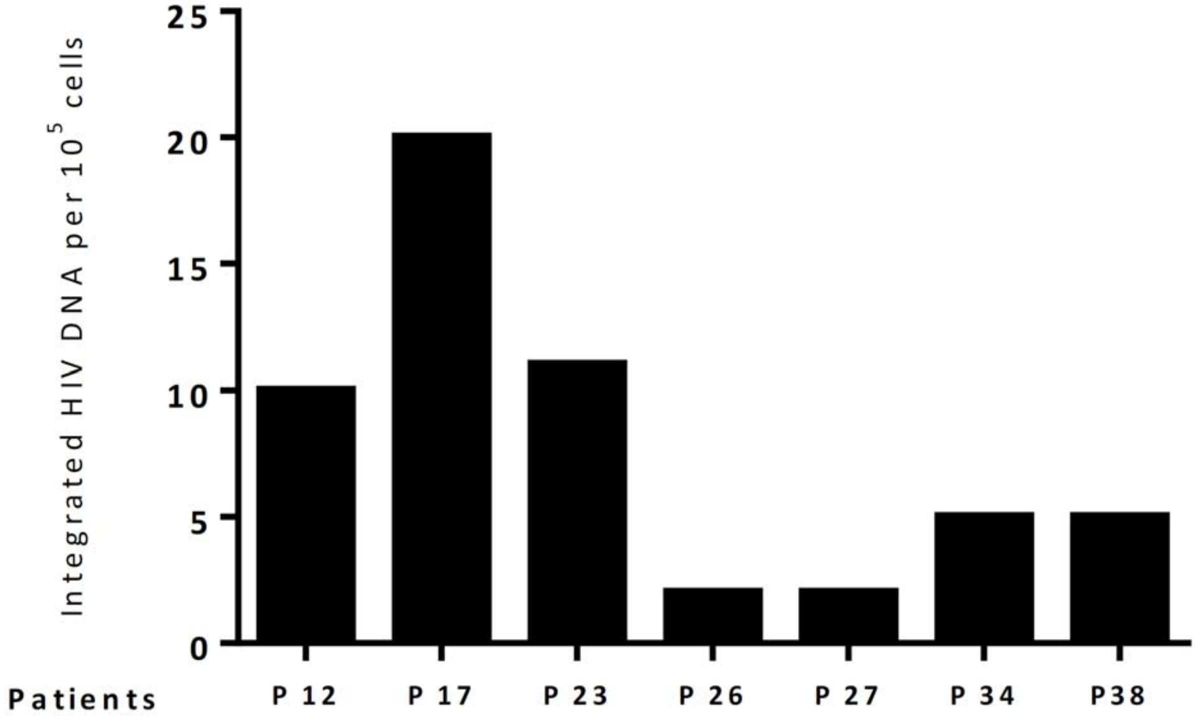

Supplementary Figure 7: Presence of integrated HIV DNA in naive CD4 T cells in patients on prolonged ART. HIV integrated DNA was detected in in sorted naive CD4 T cells ($CD45RA^{+}$ $CD45RO^{-}$ $CCR7^{+}$ $CD62L^{+}$ $CD27^{+}$ $CD95^{-}$) in 7 patients (18.4% of patients tested) (see supplementary Table 1 for the characteristics of patients).

Supplementary Figure 8

Fig. S8a

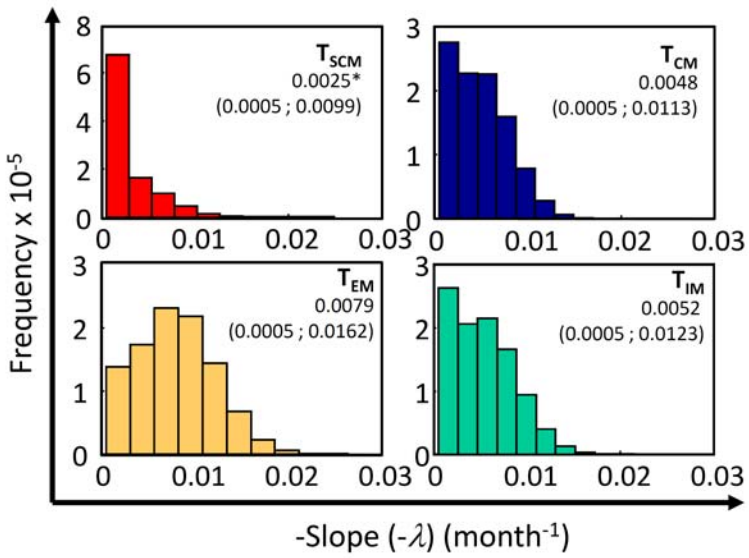

Fig S8b

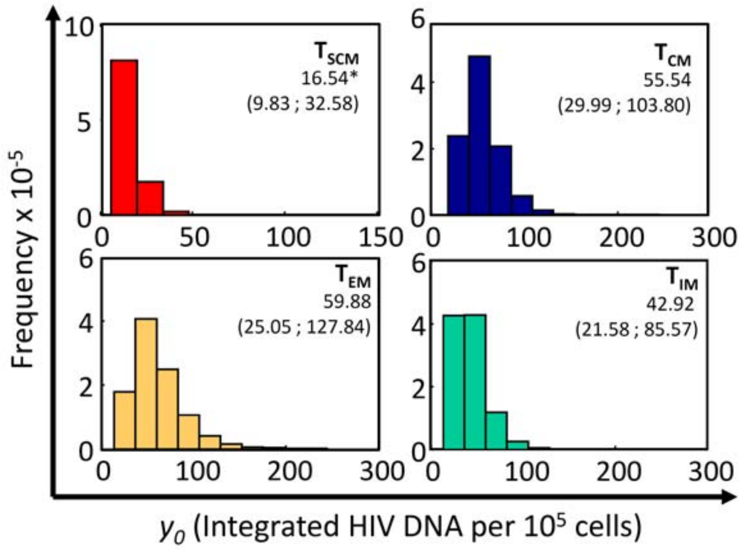

Supplementary Figure 8: Distribution of parameters λ (Fig. S8a) and y_0 (Fig. S8b) obtained by bootstrapping. λ is the value of the slope of decay (months^{-1}) and y_0 is the integrated HIV DNA copy number (per 10^5

cells) at the time of undetectable plasma virus following ART initiation. All λ values obtained by bootstrapping are negative. Positive values ($-\lambda$) are shown in **Fig.S8a**. Means and 95% confidence intervals (min ; max) of $-\lambda$ and y_0 for each memory CD4 T cell subset are also indicated. (*) indicates that the slope of decay and y_0 are significantly different between latently infected T_{SCM} and the other latently infected memory $CD4^{+}T$ cell subsets ($p < 0.05$) (for statistical analysis, see the Monte Carlo section of Methods).

Supplementary Table 1

Patients	Time on ART with undetectable plasma virus (< 40 copies /ml) (months)	Blood CD4 T cell count at time sampling (cells / mm^3)	Blood CD8 T cell count at time sampling (cells / mm^3)
P1	24	613	1014
P2	26	1705	912
P3	33	582	811
P4	35	616	807
P5	41	843	695
P6	43	778	987
P7	43	1235	1329
P8	46	745	702
P9	47	871	1128
P10	49	763	1396
P11	51	1191	1256
P12	56	1104	741
P13	63	758	1307
P14	64	713	869
P15	65	566	619
P16	71	785	594
P17	72	897	989
P18	80	550	779
P19	85	954	560
P20	85	946	753
P21	86	725	1330
P22	94	667	738
P23	94	852	453
P24	96	613	506
P25	102	933	710
P26	105	1067	869
P27	109	714	666
P28	109	840	607
P29	112	888	656
P30	117	902	1156
P31	122	1048	974
P32	127	799	814
P33	131	626	632
P34	143	614	1046
P35	144	807	888
P36	163	759	884
P37	179	784	601
P38	189	1134	1308
P39	79	856	378
P40	93	740	832
P41	70	881	538
P42	34	788	1775
P43	137	854	182
P44	69	583	1017
P45	148	807	921

Supplementary Table 1: Characteristics of the study population. Integrated HIV DNA was quantified in 38 patients (P1-P38) and virus production assay was performed in 7 patients (P39-P45). The time on ART

with undetectable plasma virus, the CD4 and CD8 T cell counts in blood and the percentages of CD4 and CD8 T cells in blood at the time of sampling are indicated for each patient.

Supplementary Methods

Proliferation assay: Sorted T_{SCM}, T_{CM}, T_{EM} and T_{IM} were stained with 0.5 µM CFSE (Invitrogen) and cultured in complete medium (RPMI with glutamax, antibiotics, pyruvate, 10% of human serum AB) with a mix of recall antigens [PPD (Statens Institut, 1 µg ml^{-1}), TT (Statens Institut, 1/50), VZV, HSV (Tebu-Bio, each 1/50) and CMV (Virusys, 1/50)] for 5 days in the presence of autologous CD8-depleted PBMC (CD8 microbeads, Miltenyi). CFSE dilution was analyzed in each subset by flow cytometry.

Virus production: Sorted resting memory CD4+ T cell subsets were obtained from patients on ART from 34 to 148 months. From 100,000 to 250, 000 sorted cells were preactivated with PHA (1 µg ml^{-1}, Sigma-Aldrich) and IL-2 (10 ng ml^{-1}, R&D Systems) for 48 hours, then cultured for 21 days in the presence of PHA-activated allogenic CD8-depleted PBMC (CD8 microbeads, Miltenyi; ratio: 1/2.5). Half of the medium was removed three times a week and replaced by fresh medium containing IL-2. HIV RNA was quantified in the supernatants by quantitative RT-PCR (Cobas Amplicor HIV-1 Detector Kit, Roche Diagnostics, lower limit of quantitation: 20 copies per ml) on day 21.

Latent HIV DNA Quantification: A first Alu-gag PCR was performed (Platinum Taq DNA Polymerase, Invitrogen) with the following primers (Eurofins MWG Operon): Alu forward primer, 5’- gCC TCC CAA ACT gCT ggg ATT ACA g- 3’; HIV gag reverse primer, 5’- gTT CCT gCT ATg TCA CTT CC- 3’.

The product of the first Alu-gag PCR was split into five 10-µL aliquots, and each aliquot was submitted to nested PCR (TaqMan Fast Advanced Master Mix, Applied Biosystems) by using the Step One Plus Real Time PCR System (Applied Biosystems). The following primers and probes were used: LTR forward primer, 5'- TTA AgC CTC AAT AAA gCT TgC C- 3'; LTR reverse primer, 5'- gTT Cgg gCg CCA CTg CTA gA- 3'; LTR wild-type probe, 5' FAM-CCA gAg TCA CAC AAC AgA Cgg gCA CA-3' TAMRA; LTR degenerated probe 1, 5' FAM- CCA gAg TCA CAT AAC AgA Cgg gCA CA-3' TAMRA; LTR degenerated probe 2, 5' FAM- CCA gAg TCA CAC AAC AgA Tgg gCA CA-3' TAMRA (all from Eurofins MWG Operon). Kinetic PCR were analyzed by the means of Step One software v2.1 (Applied Biosystems).

Quantification of GAPDH: Quantitative kinetic PCR was performed by using the following primers: Forward primer, 5' – AAA CAg CCT TgC TTg CTT Cg – 3; Reverse primer, 5' – gAC TTC CTC CAC CTg TCA GC – 3' (Eurofins MWG Operon) and the SYBR® Select Master Mix (Applied Biosystems). PCR was run on a Step One Plus Real Time PCR System (Applied Biosystems). An external scale was used to quantify GAPDH. Melting curve analysis of PCR products showed no primers dimers or non-specific PCR products that might interfere with quantitation.
